# Egypt a Residence for Invalids

**Published:** 1859-01

**Authors:** 


Egypt as a Residence for Invalids.

Dr. Dickinson, of Liverpool, being last year under the necessity of seeking to restore impaired health, visited Egypt and Nubia. On returning, he has given the results of his observations on the salubrity of these countries, in a pamphlet entitled *Egypt and Nubia; their Climate, Character, and Merits as a Winter Residence for*

*Invalids.* In ancient times, Egypt was well populated with a healthy race; and epidemic disease, if historians are to be trusted, was rare. Now, however, the reverse obtains, mainly from preventable causes. The ancient salubrity of Egypt is said to have ceased when embalming was discontinued, about the middle of the fourth century; and in Lower Egypt, the emanations from the dead, in the parts from which the overflowing of the Nile has subsided, are possibly a source of disease.

"But far more may be attributed to the great moral and physical degradation of the people, to their insufficient and unwholesome food; to their low, ill-ventilated, over-crowded, wretched huts; and the agency of animal and vegetable matters putrefying in an alluvial or marshy soil like that of the Delta."

Sanitary reform is sadly wanted in Egypt,—the more so as in itself the country possesses some of the most important elements of salubrity. Dr. Dickinson regained in it his lost health; and he encourages other invalids to follow his example. The plan to be followed is to make a well-arranged voyage up the Nile, from Cairo (lat. 30° N.) to the second cataract in Nubia (lat. 22° N.). Alexandria is not a desirable place in which to remain; the traveller should proceed at once to Cairo.

"As the northerly winds set in about November, and the climate of Cairo is then not too hot for the European invalid, he may safely arrange to arrive there at the beginning or middle of this month. The ascent of the river should be commenced before its expiration, so that Thebes may be reached in December. Here he may remain some little time, arriving in Nubia in the beginning of January, before which time the climate is too hot; and leaving it again about the middle of February for the same reason. Resting again for a short time at Thebes, the invalid should arrange to reach Cairo before the middle of March, at which time the heat on the Nile is too great to render a boat residence at all agreeable. Here he may remain until the end of the month, when the climate of Cairo itself begins to be too hot for a European invalid."

